# Severe COVID-19 Infection Associated with Endothelial Dysfunction Induces Multiple Organ Dysfunction: A Review of Therapeutic Interventions

**DOI:** 10.3390/biomedicines9030279

**Published:** 2021-03-10

**Authors:** Yujiro Matsuishi, Bryan J. Mathis, Nobutake Shimojo, Jesmin Subrina, Nobuko Okubo, Yoshiaki Inoue

**Affiliations:** 1 Department of Emergency and Critical Care Medicine, Faculty of Medicine, University of Tsukuba, Tsukuba 305-8575, Japan; nokeshimojo@yahoo.co.jp (N.S.); yinoue@md.tsukuba.ac.jp (Y.I.); 2 Pediatric Intensive Care Unit, University of Tsukuba Hospital, Tsukuba 305-8571, Japan; 3 Health & Diseases Research Center for Rural Peoples (HDRCRP), Dhaka 1205, Bangladesh; jsubrina@gmail.com; 4 Medical English Communication Center, Faculty of Medicine, University of Tsukuba, Tsukuba 305-8571, Japan; bmathis@md.tsukuba.ac.jp; 5 Neuroscience Nursing, St. Luke’s International University, Tokyo 104-0044, Japan; nobu-okubo@slcn.ac.jp

**Keywords:** endothelial dysfunction, COVID-19, ARBs, ACE2, ACE-I, bradykinin

## Abstract

Since December 2019, the SARS-CoV-2 (COVID-19) pandemic has transfixed the medical world. COVID-19 symptoms vary from mild to severe and underlying chronic conditions such as pulmonary/cardiovascular disease and diabetes induce excessive inflammatory responses to COVID-19 and these underlying chronic diseases are mediated by endothelial dysfunction. Acute respiratory distress syndrome (ARDS) is the most common cause of death in COVID-19 patients, but coagulation induced by excessive inflammation, thrombosis, and disseminated intravascular coagulation (DIC) also induce death by multiple-organ dysfunction syndrome. These associations imply that maintaining endothelial integrity is crucial for favorable prognoses with COVID-19 and therapeutic intervention to support this may be beneficial. Here, we summarize the extent of heart injuries, ischemic stroke and hemorrhage, acute kidney injury, and liver injury caused by immune-mediated endothelial dysfunction that result in the phenomenon of multi-organ dysfunction seen in COVID-19 patients. Moreover, the potential therapeutic effect of angiotensin receptor blockers and angiotensin-converting enzyme inhibitors that improve endothelial dysfunction as well as the bradykinin storm are discussed.

## 1. Introduction

SARS-CoV-2 (COVID-19) rapidly spread throughout the world from December 2019 and, within only three months, had spread to numerous nations, demonstrating high morbidity and mortality in patients with chronic diseases [1]. As of November 2020, an estimated 143,900 patients worldwide have succumbed to this pandemic [2]. COVID-19 symptoms may be mild to severe, depending on underlying chronic conditions such as pulmonary or cardiovascular diseases and the presence of diabetes, all of which induce excessive inflammatory responses to COVID-19 [3]. Interestingly, these underlying chronic diseases result in dysfunction of the endothelial cells that mediate vascular tone, causing excessive inflammation and coagulation. Acute respiratory distress syndrome (ARDS) is the most common cause of death in COVID-19 patients, but coagulation activation induced by excessive inflammation, thrombosis, and disseminated intravascular coagulation (DIC) also induces death by multiple-organ dysfunction syndrome [4,5,6]. These associations imply that maintenance of endothelial integrity is crucial for surviving COVID-19 and that therapeutic intervention to bolster these barrier cells may bring clinical benefits.

This review summarizes current knowledge of endothelial damage-mediated multi-organ dysfunction in COVID-19 patients. Moreover, we discuss the potential therapeutic effect of angiotensin receptor blockers (ARBs), ACE inhibitors (ACE-I), and related substances such as vitamin D and bradykinin for preventing endothelial dysfunction.

## 2. COVID-19-Associated Endothelial Dysfunction and Thromboinflammation

Vascular endothelium plays an important role in vascular coagulation as, normally, it prevents platelet aggregation and subsequent coagulation while promoting fibrinolysis. However, endothelial dysfunction shifts this equilibrium to thrombus formation [7]. A previous study revealed that endotheliopathy is present in COVID-19 patients and this is associated with severe status and death [8]. Excessive micro-thrombosis induced by endothelial dysfunction leads to the subsequent increase of D-dimers in severe COVID-19, which, in addition to direct viral damage, stimulates platelet aggregation and thrombosis-induced coagulopathy in the lung tissue [9]. Furthermore, subsequent “cytokine storm” induction drives not only inflammation, but also additional microcirculatory thrombosis. Indeed, two recent meta-analyses indicated that high IL–6 levels in severe disease cases [10] and at the time of hospital admission were associated with high COVID–19 mortality [11].

Viral infection induces pathogen-associated molecular patterns (PAMPs) and damage-associated molecular patterns (DAMPs), which will activate natural killer (NK) cells, macrophages, and gamma-delta T (γδ T) cells [12,13,14,15]. IFN-γ and TNF-α activated by T cells also activate subsequent pro-inflammatory cytokines while inflammatory mediators promote neutrophil release of nuclear deoxyribonucleic acid (DNA) to form neutrophil extracellular traps (NETs) that will contribute to both snaring pathogens and forming thrombi [16,17]. This process, termed immuno-thrombosis, results in a vicious cycle of inflammation and inflammation-mediated thrombosis.

Słomka et al. summarized the hematological manifestations in COVID-19 and several studies have shown hematological parameters are markers of disease severity [18]. Especially for blood coagulation, a recent study revealed that plasma von Willebrand factor antigen (VWF:Ag) and pro-coagulant factor VIII (FVIII) levels, as well as plasma VWF propeptide (VWFpp), markedly increased in COVID-19 patients [19]. Additionally, the VWFpp/VWF:Ag ratio dropped as VWF clearance was reduced and this is thought to lead to the elevated plasma VWF:Ag levels seen in severe COVID-19. Thus, VWFpp levels constitute a more sensitive and specific measure of acute EC activation [20,21] encountered in these severe COVID-19 cases [19]. Furthermore, VWF:Ag, VWF ristocetin cofactor (RCo), and VWFpp levels elevated in accordance with disease severity while levels of A Disintegrin And Metalloprotease with ThromboSpondin 1 repeats, number 13 (ADAMTS13) concordantly decreased [22]. This phenomenon indicates that such an imbalance could enhance the hypercoagulable state seen in COVID-19 patients that raises the risk of microthrombosis.

A previous study has already shown that coagulopathy plays an important role in the survival of COVID-19 patients as seen in reports that 2.0 µg/mL of D-dimer is an adequate cutoff value, with a sensitivity of 92.3% and a specificity of 83.3% for predicting in-hospital mortality (fourfold increase) upon admission [23]. Another study revealed that D-dimer values over 1.0 µg /mL were a sufficient discriminative cutoff value for asymptomatic DVT (area under the ROC curve 0.72, 95% CI 0.61–0.84) [24]. Previous systematic reviews and a meta-analysis showed that elevated D-dimer levels on admission translated to high all-cause mortality risk (odds ratio: 4.77, 95% confidence interval CI: 3.02–7.54) [25] in COVID-19 patients compared with normal D-dimer level patients, an effect confirmed by other systematic reviews and a meta-analysis [26].

## 3. Endothelial Dysfunction in Acute Respiratory Distress Syndrome (ARDS) Induced by COVID-19

The lungs are the main target of COVID-19 infection and acute lung injury (ALI) and acute respiratory distress syndrome (ARDS), characterized by injured capillary endothelium due to acute inflammatory response, are the most serious causes of COVID-19 deaths. Several vasoconstrictors and vasodilators are produced by the endothelium, such as nitric oxide (NO), endothelin-1 (ET-1), and also angiotensin-2 (Ang 2), which regulates vasomotor tone, inflammatory cells, and thrombosis and COVID-19-mediated irregularities in these vasoconstrictors and vasodilators may worsen ARDS.

ET-1, a potent endogenous vasoconstrictor [27] and reported pro-inflammatory peptide in the lungs [28], has a direct role in the development of ALI and also worsens its severity by increasing pulmonary microvasculature pressure in early sepsis [29,30]. ET-1 is mainly expressed in the lungs of patients with ARDS and increases edema while reducing the oxygenation [31]. ET-1 is also related to the pathogenesis of sepsis-induced ALI and vascular failure [32] and, from this aspect, ET blockers have been reported to have a protective effect against MODS in sepsis and ALI [33,34,35,36,37]. The reports of ET blockers for ARDS patients are still limited but Guo et al. reported a patient with influenza A (H7N9)-induced ARDS that required mechanical ventilation. After bosentan administration, right ventricular function significantly improved and weaning from mechanical ventilation occurred successfully [38].

NO is also an important vasodilator and platelet aggregation factor and NO has been reported to play roles in the pathogenesis of ALI. NO regulates microvascular permeability during ALI [39,40] and various isoforms of NO synthase (NOS), such as inducible NOS (iNOS) and endothelial NOS (eNOS), synthesize NO from L-arginine. Pulmonary vascular endothelial cells constantly generate eNOS under normal conditions [41] and a previous study reported chronic eNOS overexpression may protect the ALI by inhibiting inflammatory cytokine production [42]. However, another study reported that a high bioavailability of NO from iNOS worsens ALI [43].

Ang-II pathways are related to pathologic features of ARDS and Ang-II is not only a vasoconstrictor but also interferes with adaptive immunity through the stimulation of macrophages and other immune cells [44] in addition to enhancing inflammatory cytokines such as tumor necrosis factor-alpha (TNF-α) and interleukin (IL)-6. Ang-II mediates several pro-inflammatory responses by signaling through Angiotensin II receptor type 1 (AT1R) and the recruitment of circulating inflammatory cells to the endothelium induces an inflammatory response. This response increases leukocyte recruitment through AT1R-mediated upregulation of selectins (E-selectin and P-selectin) and chemokine (CC-chemokine ligand 5 [CCL5], known as RANTES and CC-chemokine ligand 2 [CCL2], known as MCP1) expression in endothelial cells [45,46]. These immune and inflammatory responses promote thrombosis and, interestingly, also promote microvascular COVID-19 lung vessels obstructive thromboinflammatory syndrome (MicroCLOTS) [47]. A previous case study reported that acute pulmonary embolism was present in a COVID-19 pneumonia patient and this was observed by pulmonary CT angiography [48].

These findings suggest that direct damage by viral invasion and inflammatory response, coupled with subsequent cytokine storm and endothelial dysfunction-related obstructive thrombosis, is the potential underlying mechanism for ARDS in COVID-19 patients.

## 4. COVID-19-Related Heart Effects

### 4.1. Myocardial Injury

It is well known that influenza and SARS viruses can cause myocarditis and, like these viruses, SARS-CoV-2 is associated with numerous cardiovascular complications, especially in older populations. Previous studies have revealed that endothelial dysfunction, platelet activation, excessive inflammation, and stasis are likely to induce higher risks of venous thromboembolism and acute myocardial infarctions [49]. A previous study also reported that elevated cardiac troponin T (TnT) (10%) and N-terminal pro-brain natriuretic peptides (NT-proBNP) (27.5%) were observed in COVID-19 patients [50]. An additional report noted that 27.8% of COVID-19 patients had myocardial injuries marked by elevated TnT levels and that patients with elevated TnT levels had dramatically higher mortality than normal TnT level patients (59.6% vs. 8.9%). Moreover, NT-proBNP was significantly correlated with elevated TnT levels. Not only these biomarkers, but also mortality, suggest an association between severity of myocardial injury and severity of COVID-19. Myocardial injury mostly occurs in admitted severe or fatal cases of COVID-19 and Zhao et al. summarized a previous study [51] to reveal that even mild cases may have myocardial injury (2–4%) [51,52] and severe or fatal cases have much higher rates of myocardial injury (22.2–31%) [51,53,54,55], but fatal COVID-19 cases had injury rates of approximately 28–89% [53,56,57]. Therefore, myocardial injury is an important prognostic factor in COVID-19 patients that is significantly associated with mortality [58].

### 4.2. Immune Response and Myocardial Endothelial Dysfunction

With regard to immune response effects in the myocardium, influenza virus infection increases risks of ischemic myocardial infarction (MI) and heart disease (IHD) as seen in the increase of deaths during influenza seasons [59]. Another study revealed that the number of emergency room visits was correlated with an increased number of IHD deaths among influenza-infected adults 65 years and older [60]. Increased inflammatory cytokines and cellular recruitment are thought to mediate these influenza-related cardiovascular diseases and reports of increased levels of TNFα, VEGF, GCSF, CM-CSF, basic FGF, IFNg, IL-1b, IL-1RA, IL-7, IL-8, IL-9, IL-10, IP-10, MIP-1a, MIP-1b, MCP-1, and PDGF in COVID-19 infected patients versus healthy controls bolsters this theory [51]. Interestingly, hypersensitive, TnT-related decreases in immune competency (CD4+ cells, CD8+ T cells lymphocytes, and monocytes) and increased inflammatory levels (PCT, CRP, IL-6, and neutrophils) [61] implicates an overaggressive host immune response triggering the cytokine storm that causes cardiac injury. Severely infected patients exhibit leukopenia and depleted/exhausted CD4+ and CD8+ T cells compared with moderately infected patients [62]. Indeed, several studies have reported a multi-systemic inflammatory syndrome (MIS-C), which consists of Kawasaki’s disease-like features in children with COVID-19. First observed in a few cases in the United Kingdom [63,64], another study reported that 22% of children with COVID-19 [65] exhibited this phenomenon which is mediated by cytokines and dysregulated inflammatory responses in the cardiovascular system.

## 5. COVID-19-Related Neurological Effects

### 5.1. The Routes for Covid-19 Brain Infection

Although COVID-19 has an affinity for the ACE2 receptor present in endothelial cells [66], ACE2 is also expressed in glial cells and neurons, presenting a neurotropic target for the virus [67]. Two major pathways are suspected for COVID-19 to enter the central nervous system (CNS) but the most probable is the hematogenous route. The triple-layered blood–brain barrier (BBB) prevents large molecules entering the CNS due to a highly selective structure that also excludes circulating virions. However, COVID-19 potentially infects these epithelial cells directly and the subsequent inflammation compromises the barrier function of the BBB via permeabilization, allowing infection of the CNS. A previous study observed virus-like particles in the brain capillary endothelium and active budding across endothelial cells [68].

A second pathway may be nasal as many COVID-19 patients suffer from olfactory dysfunction and this phenomenon suggests retrograde axonal transport via the olfactory bulb. The olfactory sensory neurons have a bipolar neuronal structure that connects the nasal epithelium to CNS regions and, outside of airway protection from hair and mucous membranes, is exposed to the external environment. COVID-19 rapidly penetrates into the brain via the olfactory sensory neurons and causes anosmia [69,70,71]. A recent postmortem study conducted by brain MRI revealed that COVID-19 olfactory impairment is likely to be restricted to olfactory bulbs [72] and another study found local inflammation and cytokine release as causative for injury to the olfactory sensory neurons [73].

### 5.2. A Potential Ischemic and/or Hemorrhagic Stroke Mechanism

It has been reported that 36.4% of patients with COVID-19 at hospitals in Wuhan, China experienced neurological manifestations and acute cerebrovascular disease more frequently observed in severe COVID-19 patients [74]. An additional study showed that 4.6% of patients with COVID-19 developed acute ischemic stroke, with half of those incidents classified as large vessel occlusions [75], while a UK report found that all instances of COVID-19-mediated acute ischemic stroke were large vessel occlusions [76], a phenomenon verified by a U.S. study [77]. Taken together, these global reports indicate that large vessel occlusions are the most frequent complication of acute ischemic stroke in COVID-19 patients.

Generally, ischemic stroke is caused by cardioembolism and this is in line with reports of arrhythmia during COVID-19 infection. A current study reported that 16.7% of admitted COVID-19 patients had arrhythmia [52] while another study reported that 5.9% of COVID-19 patients had either ventricular fibrillation (VF) or ventricular tachycardia (VT) [78]. This type of arrhythmia and excessive micro thrombosis induced by endothelial dysfunction could explain the subsequent increase in stroke risk seen in severe COVID-19 cases.

There are relatively few reports, but hemorrhagic stroke has also occurred. Recent studies indicate that COVID-19 affects both the CNS and peripheral nervous systems (PNS) [79,80] with a phenomenon newly dubbed “Neuro-COVID”. The European Neurocritical Care Society (NCS) and Academy of Neurology (EAN) has endorsed the Global Consortium Studies of Neurological Dysfunction in COVID-19 (GCS-Neuro-COVID) and thus has established a formal collaboration [81] for consensus and harmonization of data. Planned and current studies will continue to shed light on COVID-related neurological dysfunction of all kinds and possibly highlight interventional strategies for dealing with COVID-specific neurological sequelae.

### 5.3. COVID-19 Related Kidney Injury

Several studies have already reported associations between COVID-19 and acute kidney injury (AKI) [3,82,83] where factors of kidney involvement on tests such as urea nitrogen, serum creatinine, and/or proteinuria, as well as other evidence of AKI, were independently associated with hospital death [3]. COVID-19 induced kidney dysfunction usually manifests as increased urea nitrogen levels and blood creatinine [84,85] resulting from tubular damage and impaired glomerular filtration. High ACE2 expression is found in the proximal tubular epithelial cells whereas weak to moderate signals are located in Henle’s loop, collecting ducts, glomeruli, and the distal tubules thought to be a potential target for kidney injury [86,87,88,89,90,91,92]. Cellular transmembrane serine proteases (TMPRSSs) and ACE2 are co-expressed in proximal straight tubule cells and podocytes, indicating that the kidney cells are vulnerable to COVID-19 infection [93]. Pathological changes may also be observed and severe acute tubular necrosis was visible upon pathological light microscopic examination during autopsies of six patients who had died from COVID-19 in Wuhan [94].

Importantly, 32 out of 33 COVID-19 patients who developed AKI did not survive [95] and this significant association indicates that AKI is an important prognostic factor in COVID-19 that progresses to mortality. The prevalence of AKI is not consistently reported but a previous systematic review and a meta-analysis based on 21,531 patients found an AKI prevalence of 12.3% and 5.4%, respectively, in COVID-19 patients taking renal replacement therapy (RRT), but regionality may be a factor as AKI incidence was lower in Asia (6.9%) compared to Europe (22.9%) and North America (34.6%). Moreover, ICU admission reflected very high rates of AKI (39.0%) and RRT use (16.3%) [84]. Another study reported that AKI incidence in critically ill COVID-19 patients in the ICU was 29% [53], while patients with kidney insufficiencies marked by abnormal urea nitrogen, serum creatinine, and proteinuria were at increased risk of hospital death [3]. Another study of 5700 patients revealed 81 patients needed renal replacement therapy after admission and patients with diabetes were more likely to suffer from AKI [96].

Continuous renal replacement therapy (CRRT) might block the cytokine storm and improve the prognosis of COVID-19 patients. However, a previous study already reported that CRRT filters quickly clog due to coagulopathy and therefore adequate anticoagulation therapy will be needed for COVID-19 patients, especially severe cases, but specific knowledge of best practices in these cases is still limited [25].

### 5.4. COVID-19-Related Liver Injury

**Figure 1:**
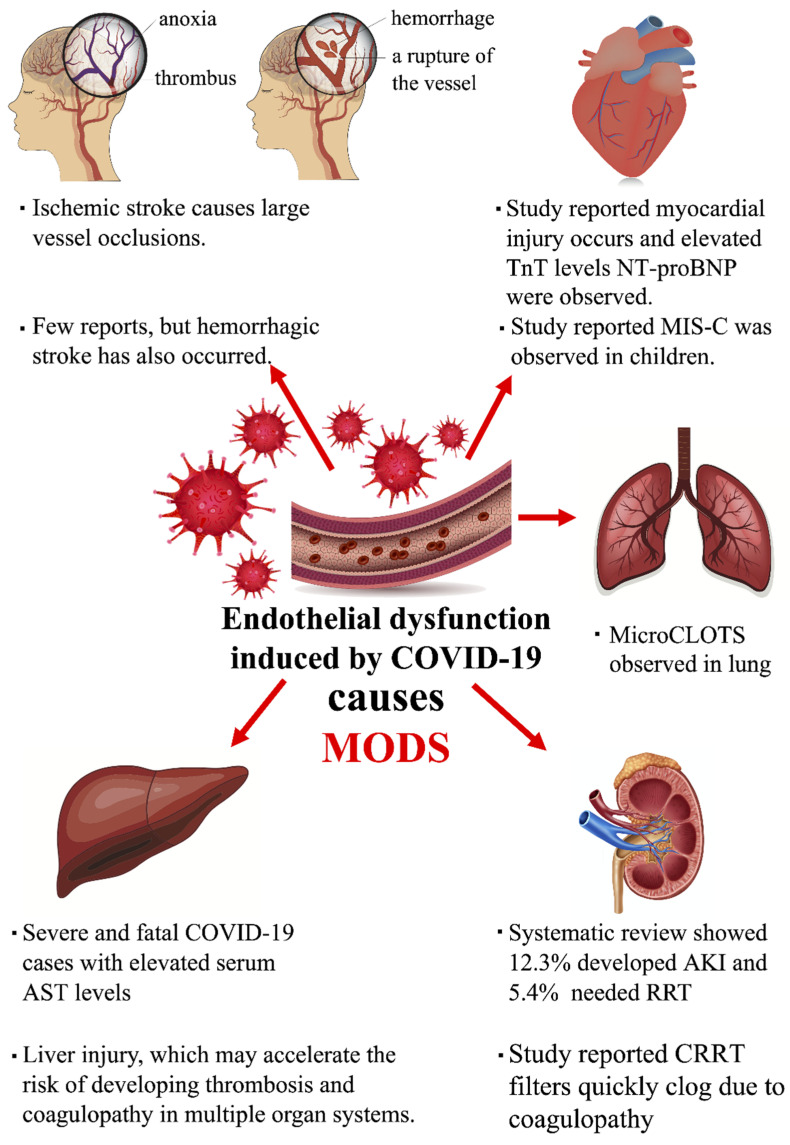
Endothelial dysfunction induced by COVID-19 develops into MODS.

Clinical details of liver injury specifics associated with COVID-19 have yet to be reported. However, a generally overactive immune response induced by COVID-19 infection and subsequent dysregulated inflammatory cellular responses (cytokine storm) may lead to liver injury [97]. A previous study has already reported liver enzyme abnormalities, elevated serum alanine aminotransferase (ALT), and aspartate aminotransferase (AST) in 43.4% of surveyed COVID-19 patients [3], an effect also reported in a systematic review and a meta-analysis of severe and fatal COVID-19 cases where elevated serum AST levels were found. This association implies that severe COVID-19 patients are at risk of liver injury, which may accelerate the risk of developing vascular thrombosis [98], coagulopathy, and subsequent clotting issues in multiple organ systems. Patients with comorbid fatty liver or other hepatic diseases may thus be at risk of developing severe or fatal COVID-19 infections and should be monitored closely for signs of liver damage. We summarized the commodity of multi-organ dysfunction by endothelial dysfunction in COVID-19 patients in Figure 1.

### 5.5. Treatment of COVID-19-Associated Endothelial Dysfunction

There are several studies that have investigated the therapeutic effect of renin angiotensin system (RAS) inhibitors and statins. Angiotensin II (Ang II), an endogenous peptide hormone, is the main effector of the RAS for homeostatic adjustment of pressure in the cardiovascular system. Gencer et al. shed light on the significant role of RAS in COVID-19 in a review [99]; however, using ARB and ACE-inhibitors itself is controversial within the literature [51,100,101,102,103,104,105,106].

Two viewpoints exist, the first being that ARB and ACE inhibitors upregulate ACE2 expression, which makes it easy for SARS-CoV-2 virions to enter target cells. As angiotensin-converting enzyme 2 (ACE2), originally discovered in 2000 by two researchers [107,108], is ubiquitous throughout the heart and kidneys, increased ACE2 expression by these regulators may increase the infective load of SARS-CoV-2 in the cardiovascular system. Therefore, it is hypothesized that increasing ACE2 via ARB and ACE inhibitors is contraindicated for COVID-19 patients [109].

The second viewpoint is that ARB and ACE inhibitors enhance vasodilation and anti-inflammatory effects for COVID-19 patients, which is of importance due to associations between Ang II and reactive oxygen species (ROS). Previous studies have hypothesized that Ang II enhances ROS generation in mitochondria via the “ROS-induced ROS release” (RIRR) mechanism [110,111] and the NAD(P)H oxidase-derived ROS stimulated by Ang II may lead to a mitochondrial ROS burst that precipitates cellular death on a multicellular scale [112]. Several systematic reviews and meta-analyses revealed that ACE inhibitors, in addition to angiotensin receptor blockers (ARBs), have been shown to improve endothelial dysfunction by flow-mediated vasodilatation (FMD), a widely-used method for evaluate endothelial function [113,114]. This viewpoint thus asserts that the generation of ROS, which has both primary (tissue damage) and secondary (signaling) is an important component of the vascular damage seen in COVID-19 and that ARB and ACE inhibitors, although freeing up ACE2, dampen ROS that causes microcapillary and intimal damage within the vascular system.

However, for the pulmonary system, Imai et al. evaluated the role of ACE2 and Ang II in lung injury by using a septic mouse model. As the ACE2-knockout mice had more severe forms of lung injury, the conclusion was that ACE2 is overall protective in sepsis [115]. This data indicate that ACE2, although vital for COVID-19 penetration into somatic and neural cells, may provide a measure of protection by mediating ROS levels in the vascular and pulmonary systems. More studies will need to be conducted that examine the mechanistic interplay and how ARB and ACE inhibitors disrupt that balance.

### 5.6. Other Potential Drug Therapies for Treatment of COVID-19

Although the effectiveness is controversial, there are several drug therapies currently under examination for COVID-19. Hydroxychloroquine (HCQ) and chloroquine (CQ), used in the treatment of uncomplicated malaria, were first reported from France in June 2020 and that study administered hydroxychloroquine and azithromycin (HCQ-AZ) for 26 patients with COVID-19, resulting in 20 patients showing a significant reduction of viral loads [116]. Since that study had a small sample size, another study carried out in the USA analyzed the therapeutic effect of hydroxychloroquine in 1446 consecutive patients and could not reveal any significant therapeutic effects of hydroxychloroquine [116]. However, another French study confirmed the therapeutic effect of HCQ-AZ by analyzing 3737 patients with COVID-19, including 3119 (83.5%) treated with HCQ-AZ [117]. Other retrospective observational studies [118,119] and a prospective observational study [120], as well as RCTs [121,122], confirmed the therapeutic effect of HCQ and CQ.

Based on these studies [118,119,120,121,122], the U.S. Food and Drug Administration (FDA) approved emergency use authorization (EUA) of HCQ and CQ for COVID-19 in March 2020 but revoked it in June 2020 because of perceived limited effectiveness [123]. Additionally, side effects of these drugs have been reported, especially increased risk for Long QT Syndrome (LQTS), and several studies have confirmed this phenomenon [124,125].

Anti-inflammation therapy has also been reported as an effective treatment for COVID-19. Colchicine, an anti-inflammatory and immunomodulatory agent, was suggested to prevent some complications of COVID-19 by inhibiting IL-1 production. Several RCTs have since demonstrated this efficacy such as the GRECCO-19 (the Greek Study in the Effects of Colchicine in COVID-19 Complications Prevention) trial that evaluated a total of 105 patients and reported significantly improved clinical outcomes [126]. The ongoing COLCOVID (The ECLA PHRI COLCOVID Trial: Effects of Colchicine on Moderate/High-risk Hospitalized COVID-19 Patients) trial (ClinicalTrials.gov: NCT04328480) has recruited 2500 patients, but the COLCORONA (Colchicine Coronavirus SARS-CoV2) multi-center RCT to evaluate the efficacy of colchicine in high-risk adult patients with COVID-19 (ClinicalTrials.gov: NCT04322682) was recently finished and briefly reported that colchicine reduced hospitalizations by 25%, the need for mechanical ventilation by 50%, and deaths by 44% [127].

Dexamethasone, a synthetic corticosteroid, has also been suggested for decreasing COVID-19 mortality as corticosteroids have been extensively used for severe lung conditions such as Acute Respiratory Distress Syndrome (ARDS) and previous outbreaks of SARS and Middle East respiratory syndrome (MERS) [128,129]. The RECOVERY (Randomized Evaluation of COVid-19 thERapY) trial revealed that the use of dexamethasone reduced 28-day mortality for oxygen therapy in mechanical ventilation patients [130] and also initially attempted to judge the efficacy of HCQ but suspended recruiting for this purpose after no significant benefits were observed [131].

### 5.7. Bradykinin as a Potential Target Protein for COVID-19

Recently, Garvin et al. reported an in silico analysis of the potential relationship between severity of COVID-19 and bradykinin [132]. Generally, ACE2 is the main receptor for COVID-19 and is not highly expressed in lungs. However, as gene expression analysis of bronchial lavage samples showed the dramatic upregulation of ACE2 (199-fold), AGTR1 (430-fold), and AGTR2 (177-fold) receptors with downregulation of ACE (8-fold), compared to controls, this phenomenon indicates a shift in the RAS system to produce Ang1–9. Genetic pathway analysis also detected the two networks extensively involved in vasoconstriction and contained, among others, ACE, AGTR1, Bradykinin Receptor 2 (BDKR2), NOS1, and NOS2. Additionally and extensively involved in vasodilation, increased vascular permeabilization, and altered fluid balance, were ACE2, AGTR2, and the Vitamin D Receptor (VDR). Vitamin D, already known to reduce acute lung injury by blocking the Ang-2-Tie-2- myosin light chain (MLC) kinase cascade and the renin-angiotensin system [133], mediates the expression of ACE2 [134]. As the SARS-CoV-2-enhanced expression of ACE2 could theoretically increase viral entry into cells, vitamin D may seem counterintuitive but Murai et al. did significant work to evaluate the therapeutic effect of vitamin D for COVID-19. Random administration of vitamin D (200,000 IU) or placebo for 240 hospitalized patients with moderate to severe COVID-19 (non-ventilator status) [135] showed a trend for a therapeutic effect of vitamin D for COVID-19 but this was not significant. However, the editorial mentioned the importance of remaining open-minded to emerging results from rigorously conducted studies of vitamin D [136].

Bradykinin is responsible for the vasodilation and permeability of the pulmonary vascular system. The kinin-kallikrein system is a zymogen system that, after activation, leads to the release of bradykinin. Binding bradykinin to BDKR2 increases vascular permeability and subsequent pulmonary edema [137]. Imai et al. reported that ACE2 plays an important role for preventing ALI [115] but, unusually, they also reported no differences in hydrostatic pressure compared with ACE2 knockout and controls, concluding that severe lung edema in ACE2 knockout mice does not seem to be secondary to systemic hemodynamic alterations [115]. Unlike the RASS system, bradykinin controls permeability and vasodilatation without vasoconstriction. Thus, this phenomenon can be explained by the kinin-kallikrein system. Bradykinin is only a substrate for ACE, not a substrate for ACE2 [138,139]. Therefore, ACE2 does not inactivate bradykinin but ACE2 and bradykinin do cross talk through potent ligands of BDKR1 in the lung, namely des-Arg9-BK and Lys des-Arg9-BK [138].

There are still limited clinical reports describing the associations between bradykinin and ARDS since these patients demonstrate markedly elevated levels of bradykinin in the bronchoalveolar lavage fluid (BALF) samples compared with healthy controls [140].

From these data, blocking bradykinin B1 receptors (BKB1R) and bradykinin B2 receptors (BKB2R) may be a potential therapeutic target. However, there is no licensed BKB1R drug to date [141] and the BKB2R drug icatibant (Firazyr) is only available in the U.S. and Europe. There are already reports on the use of icatibant for COVID-19 patients as van de Veerdonk et al. included 10 patients for treatment with three doses of 30 mg of icatibant by subcutaneous injection at 6-h intervals and evaluated the therapeutic effect by matched-pair analysis [142]. All nine patients prescribed icatibant experienced a marked decrease in oxygen supplementation and four patients (44%) were weaned from oxygen within 10–35 h after three doses of icatibant. Additionally, eight out of nine patients (89%) treated with icatibant confirmed a reduction in oxygen use of 3 L/min or greater after 24 h. After matched-pair analysis (nine patients using icatibant vs. 18 control patients), only three out of 18 patients (17%) without icatibant showed a reduction in oxygen use of 3 L/min or greater after 24 h. From these results, the authors revealed an association between the use of icatibant and improvements in lung injury. Interestingly, three out of 10 patients (30%) using icatibant did need to go back on oxygen again, which may be due to icatibant’s short-acting effect (half-life of 2 h) [143]. Recently, an RCT to evaluate the efficacy and safety of icatibant, a C1 esterase/kallikrein inhibitor, in severe COVID-19 has been launched [144] and this study may lead to a more rigorous association between COVID-19 infection and bradykinin systems.

## 6. Future Direction

ARB and ACE-I usage in COVID-19 is still controversial as observational studies cannot eliminate bias and confounding factors. Recent reviews stated that underlying diseases such as hypertension are not the sole independent risk factor and ARB and/or ACE-I are also not risk factors [145]. However, a previous review noted a therapeutic effect of ARB for endothelial dysfunction during viral infection and subsequent sepsis [145] while a recent review on COVID-19 treatment also confirmed the therapeutic effect of ARB for endothelial dysfunction [146]. Such conflicting reports currently complicate the removal of bias in results, but Donald B. Rubin has suggested expanding the method of causal inference to reveal any rigorous associations between factors and results in observational studies [147].

Currently, several randomized clinical trials (RCTs) are ongoing to confirm the therapeutic effect of ARB and ACE-I for COVID-19 as shown in Table 1. Diverse studies of various sizes in adults plus one study in the elderly are awaiting study completion and publication. The COVID-RASi Trial (COVID-19) focuses exclusively on elderly patients with hypertension (HT), diabetes mellitus (DM), coronary artery disease (CAD), history of myocardial infarction (MI), heart failure (HF) ischemic stroke, or renal dysfunction. This RCT compares the mortality of relatively high-risk patients after ACE-I/ARB treatment and standard care. Meanwhile, the REPLACE COVID (Randomized Elimination and Prolongation of ACE inhibitors and ARBs in coronavirus 2019) trial, the Randomized ACORES-2 Study (ACE Inhibitors or ARBs Discontinuation for Clinical Outcome Risk Reduction in Patients Hospitalized for the Endemic Severe Acute Respiratory Syndrome Coronavirus SARS-CoV-2 Infection), the ACEI-COVID (Stopping ACE-inhibitors in COVID-19) trial, the BRACE CORONA trial (Continuing versus suspending angiotensin-converting enzyme inhibitors and angiotensin receptor blockers: Impact on adverse outcomes in hospitalized patients with severe acute respiratory syndrome coronavirus 2 SARS-CoV-2), and RASCOVID-19 trial (Effects of Discontinuing Renin-angiotensin System Inhibitors in Patients With COVID-19) are being conducted to compare outcomes from continuing or discontinuing use of ARB or ACE-I treatment. On the other hand, CAPTOCOVID (Efficacy of Captopril in Covid-19 Patients With Severe Acute Respiratory Syndrome (SARS) CoV-2 Pneumonia), CLARITY (Controlled evaLuation of Angiotensin Receptor Blockers for COVID-19 respIraTorY Disease), and STAR-COVID (Telmisartan in Respiratory Failure Due to COVID-19) trials are currently comparing the outcomes of using or foregoing ARB or ACE-I treatments. Additionally, angiotensin 1–7 is being investigated as a therapeutic treatment for COVID-19 in two RCTs (ClinicalTrials.gov Identifier: NCT04332666: NCT04605887).

Moreover, a living network meta-analysis through the Cochrane Library is now active to rate interventions for the treatment of COVID-19 [148]. These living network meta-analyses have recently been suggested as the new paradigm in comparative effectiveness research [149,150] as they can reach robust conclusions on the relative effectiveness of treatments earlier than sequential meta-analyses, reducing research waste, and offering timely recommendations [149,150,151]. Moreover, even though the idea of the bradykinin storm is now gaining attention, no RCTs have been done or are being done to confirm the therapeutic effect. As supercomputers were able to detect the potential relationship between the bradykinin storm and COVID-19, both in silico and living network meta-analyses may synergistically and systematically detect targets. As recent third waves of infection have been announced, time is of the essence and these rapid data sorting and evaluation methods may be invaluable for determining best practice in treating both severe and “long-haul” cases of COVID-19 that feature cardiovascular, neurological, and multi-organ sequelae.

## 7. Conclusions

Based on current evidence, COVID-19 infections cause frequent multi-organ damage, mainly via endothelial dysfunction in the heart, brain, kidney, and liver. Usage of ARB and ACE-I, potential tools to ameliorate this endothelial dysfunction, have competing theories and current RCTs will be the ultimate judge of their usefulness in both severe and chronic cases of COVID-19. While several studies have implicated that bradykinin is a promising target protein for COVID-19, the results of the various clinical trials for ARB, ACE I, and bradykinin blockers must be tabulated and analyzed for rigorous associations. Further studies, especially well constructed RCTs, will bring clear evidence for definitive COVID-19 treatment guidelines.

## Figures and Tables

**Table 1:** Ongoing RCTs to confirm the therapeutic effect of ARB and ACE-I for COVID-19.

No.	Country	Title	Sample Size	Age	Subjects	ARB or ACE-I	Drug	Dose	Method	Masking	Primary Outcome	Start Date	Estimated Study Completion Date
1	Sweden	Using the blood pressure medication losartan to improve outcomes for patients with SARS CoV-2	750	Age > 18 years	GCS ≥ 14	ARB	Losartan	Maximum of 100 mg	Standerd care	Open label	1. All-cause mortality at day 28 from randomization 2. Occurrence of ICU admission during hospital stay. 3. Need for and duration of MV. 4. Peak level and area under the curve during hospitalization for National Early Warning score 2 (NEWS2) score. 5. Peak level and area under the curve during hospitalization for CRP score	March, 2020	February, 2021
2	USA	Elimination or Prolongation of ACE Inhibitors and ARB in Coronavirus Disease 2019 (REPLACECOVID)	152	Age > 18 years	Current Use of ARB or ACE-I	ARB or ACE-I	Not specific	Not specific	Continuation compared with discontinuation of ARB or ACE-I	Single (Participant)	1. Time to death. 2. The number of days supported by MV or ECMO. 3. the number of days supported by RRT or pressor/inotropic therapy. 4. Modified SOFA score.	March, 2020	December, 2020
3	USA	Do Angiotensin Receptor Blockers Mitigate Progression to Acute Respiratory Distress Syndrome With SARS-CoV-2 Infection	200	Age > 18 years	Mild to moderate respiratory symptoms of COVID-19	ARB	Losartan	12.5mg (investigator has option to increase dose on days 2–10 based on tolerance of SBP)	Standard of Care	None (Open Label)	1. MV days	March, 2020	December, 2020
4	Paris	ACE Inhibitors or ARBs Discontinuation for Clinical Outcome Risk Reduction in Patients Hospitalized for the Endemic Severe Acute Respiratory Syndrome Coronavirus (SARS-CoV-2) Infection: the Randomized ACORES-2 Study	554	Age > 18 years	Current Use of ARB or ACE-I	ARB or ACE-I	Not specific	Not specific	Continuation compared with discontinuation of ARB or ACE-I	None (Open)	1. Improvement of two points on a seven-category ordinal scale. 2. Live discharge from the hospital	April, 2020	August, 2020
5	Ireland	Coronavirus (COVID-19) ACEi/ARB Investigation (CORONACION)	2414	Age > 60 years	Current use of ARB or ACE-I for the treatment of hypertension	ARB or ACE-I	Not specific	Not specific	Alternate anti-hypertensive medication (Thiazide, Thiazide-like diuretics or Calcium Channel Blockers)	None (Open Label)	1. Number of deaths. 2. Number of MV. 3.Require hospitalization for non-invasive ventilation (NIV)	April, 2020	December, 2021
6	Netherlands	Valsartan for Prevention of Acute Respiratory Distress Syndrome in Hospitalized Patients With SARS-COV-2 (COVID-19) Infection Disease	651	Age > 18 years	Hospitalization for COVID-19	ARB	Valsartan	Dosages will be titrated to blood pressure with a maximum of 160mg	Placebo	Quadruple (Participant, Care Provider, Investigator, Outcomes Assessor)	1. First occurrence of ICU admission. 2. MV or all-cause mortality	April, 2020	December, 2021
7	Austria	Stopping ACE-inhibitors in COVID-19 (ACEI-COVID)	208	Age > 18 years	Current Use of ARB or ACEI	ARB or ACE-I	Not specific	Not specific	Continuation compared with discontinuation of ACEI or ARB	Open label	1. SOFA Score. 2. Admission to ICU. 3. Use of mechanical ventilation. 4. All-cause mortality	April, 2020	May, 2022
8	Brazil	Continuing versus suspending angiotensin-converting enzyme inhibitors and angiotensin receptor blockers: Impact on adverse outcomes in hospitalized patients with severe acute respiratory syndrome coronavirus 2 (SARS-CoV-2)--The BRACE CORONA Trial	500	Age > 18 years	Current Use of ARB or ACEI	ARB or ACE-I	Not specific	Not specific	Continuation compared with discontinuation of ACEI or ARB	Not writen	1. Mortality 2. Discharge days	April, 2020	Not writen
9	Austria	Discontinuation of ACE inhibitors in patients with COVID-19 infection	798	Age > 18 years	Current Use of ARB or ACEI	ARB or ACE-I	Not specific	Not specific	Continuation compared with discontinuation of ACEI or ARB	Not written	1. Maximum SOFA scores. 2. Mortality	April, 2020	Not writen
10	Netherlands	A clinicial trial to investigate the effect of valsartan compared to placebo on acute respiratory failure in hospitalized SARS-CoV-2-infected patients	651	Age > 18 years	Not specific	ARB	Valsartan	80–160 mg	Placebo-controlled	Double-blind	1. ICU admission. 2. MV. 3.Mortality	April, 2020	Not writen
11	Germany	Treatment of Sars-CoV2 infections (Covid-19) in patients without or with chronic kidney disease (CKD) with valsartan vs placebo, a three-armed randomized, partly blinded trial	300	Age > 18 years	Pre-existing chronic renal insufficiency in any degree of severity	ARB	Valsartan	80mg	Study patients randomly converted from ACE Inhibitors to ARB	Partly blinded	1. Severity rating on a 7-point scale. 2. Hospital discharge	April, 2020	Not writen
12	France	COVID-19—ACORES-2 study: ACE inhibitors or ARBs discontinuation for Clinical Outcome Risk reduction in patients hospitalized for the Endemic Severe acute respiratory syndrome coronavirus (SARS-CoV-2) infection	554	Age > 18 years	Non-ICU	ARB or ACE-I	Not specific	Last prescription prior to admission	Continuation compared with discontinuation of ACEI or ARB	Not written	1. Improvement of two points on a seven-category ordinal scale. 2. Live discharge from the hospital	April, 2020	Not writen
13	Denmark	Effects of Discontinuing Renin-angiotensin System Inhibitors in Patients With COVID-19 (RASCOVID-19)	215	Age > 18 years	Current Use of ARB or ACEI	ARB or ACE-I	Not specific	Not specific	Continuation compared with discontinuation of ACEI or ARB	Single (Outcomes Assessor)	1. Days alive and out of hospital	May, 2020	December, 2020
14	Paris	Efficacy of Captopril in Covid-19 Patients With Severe Acute Respiratory Syndrome (SARS) CoV-2 Pneumonia (CAPTOCOVID)	230	Age > 18 years	Acute respiratory failure requiring oxygen administration ≥ 3L/mn	ACE-I	Captopril	25mg nebulization	Standard care	None (Open)	1. MV-free survival	May, 2020	August, 2020
15	Argentina	Telmisartan for Treatment of COVID-19 Patients	400	Age > 18 years	Hospitalization for COVID-19	ARB	Telmisartan	160mg	Standard care	None (Open)	1. CRP	May, 2020	October, 2020
16	Australia	Controlled evaLuation of Angiotensin Receptor Blockers for COVID-19 respIraTorY Disease (CLARITY)	605	Age > 18 years	Non ARB or ACE-I user	ARB	Not specific	Not specific	Standard care	Single (Outcomes Assessor)	1.7-Point National Institute of Health Clinical Health Score.	June, 2020	January, 2021
17	Germany	Treatment of Sars-CoV2 infections (Covid-19) with valsartan vs placebo, a three-armed randomized, partly blinded trial	300	Age > 18 years	Pre-existing chronic renal insufficiency in any degree of severity	ARB or ACE-I	Valsartan	80 mg	Three arm	Partly blinded	1. Severity rating on a 7-point scale	June, 2020	Not writen
18	Mexico	Telmisartan in Respiratory Failure Due to COVID-19 (STAR-COVID)	60	Age > 18 years	Expected ICU stay of > 48 h	ARB	Telmisartan	Receive telmisartan 40 mg daily plus standard care.	Standard care	None (Open)	1. Mortality	August, 2020	March, 2021
19	Netherlands	Angiotensin-(1,7) Treatment in COVID-19: the ATCO Trial (ATCO)	60	Age > 18 years	Expected ICU stay of > 48 h	Other	Angiotensin 1–7	0.2 mcg/Kg/h for 48h	Standard care	Double-blind	1. Ventilator- free days	September, 2020	June, 2021
20	Canada	The COVID-RASi Trial (COVID-19)	1155	65 Years and older	HT, DM, CAD, history of MI, HF, ischemic stroke or renal dysfunction.	ARB and ACE-I	Three arms: Standerd care, ACE-I treatment and ARB treatment.	Dose adjustments as appropriate.	Standard care	Single (Outcomes Assessor)	1. Mortality. 2. MV. 3. ICU admission	October, 2020	August, 2022
21	Israel	Angiotensin 1–7 as a Therapy in the Treatment of COVID-19	120	Age > 18 years	Moderate lung status (without MV)	Other	Angiotensin 1–7	500 mcg/kg /day	Placebo	Triple (Participant, Care Provider, Investigator)	1. Need for MV. 2. Mortality.	November, 2020	April, 2024

ACE-I: Angiotensin-converting-enzyme inhibitor, ARB: Angiotensin II Receptor Blocker, CAD: Coronary artery disease, CRP: C-reactive protein, DM: Diabetes mellitus, ECMO: Extracorporeal membrane oxygenation, HF: Heart failure, HT: Hyper tension, ICU: Intensive care unit, MI: Myocardial infarction, MV: Mechanical Ventilation, RRT: Renal replacement therapy, SBP: Systolic blood pressure, SOFA: Sequential organ failure assessment score.

## Data Availability

This study contains no original data.

## References

[1] Connors J.M., Levy J.H. (2020). COVID-19 and its implications for thrombosis and anticoagulation. Blood.

[2] Number of Novel Coronavirus (COVID-19) Deaths Worldwide as of 27 November 2020, by Country. https://www.statista.com/statistics/1093256/novel-coronavirus-2019ncov-deaths-worldwide-by-country/.

[3] Chen N., Zhou M., Dong X., Qu J., Gong F., Han Y., Qiu Y., Wang J., Liu Y., Wei Y. (2020). Epidemiological and clinical characteristics of 99 cases of 2019 novel coronavirus pneumonia in Wuhan, China: A descriptive study. Lancet.

[4] Mehta P., McAuley D.F., Brown M., Sanchez E., Tattersall R.S., Manson J.J. (2020). COVID-19: Consider cytokine storm syndromes and immunosuppression. Lancet.

[5] Jose R.J., Manuel A. (2020). COVID-19 cytokine storm: The interplay between inflammation and coagulation. Lancet Respir. Med..

[6] Wright D.J.M. (2020). Prevention of the cytokine storm in COVID-19. Lancet Infect. Dis..

[7] Yau J.W., Teoh H., Verma S. (2015). Endothelial cell control of thrombosis. BMC Cardiovasc. Disord..

[8] Goshua G., Pine A.B., Meizlish M.L., Chang C.H., Zhang H., Bahel P., Baluha A., Bar N., Bona R.D., Burns A.J. (2020). Endotheliopathy in COVID-19-associated coagulopathy: Evidence from a single-centre, cross-sectional study. Lancet Haematol..

[9] Keith P., Day M., Choe C., Perkins L., Moyer L., Hays E., French M., Hewitt K., Gravel G., Guffey A. (2020). The successful use of therapeutic plasma exchange for severe COVID-19 acute respiratory distress syndrome with multiple organ failure. SAGE Open Med. Case Rep..

[10] Zeng F., Huang Y., Guo Y., Yin M., Chen X., Xiao L., Deng G. (2020). Association of inflammatory markers with the severity of COVID-19: A meta-analysis. Int. J. Infect. Dis..

[11] Tian W., Jiang W., Yao J., Nicholson C.J., Li R.H., Sigurslid H.H., Wooster L., Rotter J.I., Guo X., Malhotra R. (2020). Predictors of mortality in hospitalized COVID-19 patients: A systematic review and meta-analysis. J. Med. Virol..

[12] Chousterman B.G., Swirski F.K., Weber G.F. (2017). Cytokine storm and sepsis disease pathogenesis. Semin. Immunopathol..

[13] Vardhana S.A., Wolchok J.D. (2020). The many faces of the anti-COVID immune response. J. Exp. Med..

[14] Akira S., Uematsu S., Takeuchi O. (2006). Pathogen recognition and innate immunity. Cell.

[15] Medzhitov R. (2007). Recognition of microorganisms and activation of the immune response. Nature.

[16] Mangalmurti N., Hunter C.A. (2020). Cytokine Storms: Understanding COVID-19. Immunity.

[17] Del Turco S., Vianello A., Ragusa R., Caselli C., Basta G. (2020). COVID-19 and cardiovascular consequences: Is the endothelial dysfunction the hardest challenge?. Thromb. Res..

[18] Słomka A., Kowalewski M., Żekanowska E. (2020). Coronavirus disease 2019 (COVID–19): A short review on hematological manifestations. Pathogens.

[19] Ward S.E., Curley G.F., Lavin M., Fogarty H., Karampini E., McEvoy N.L., Clarke J., Boylan M., Alalqam R., Worrall A.P. (2020). Von Willebrand factor propeptide in severe coronavirus disease 2019 (COVID-19): Evidence of acute and sustained endothelial cell activation. Br. J. Haematol..

[20] Hollestelle M.J., Donkor C., Mantey E.A., Chakravorty S.J., Craig A., Akoto A.O., O’Donnell J., Van Mourik J.A., Bunn J. (2006). von Willebrand factor propeptide in malaria: Evidence of acute endothelial cell activation. Br. J. Haematol..

[21] van Mourik J.A., Boertjes R., Huisveld I.A., Fijnvandraat K., Pajkrt D., van Genderen P.J., Fijnheer R. (1999). von Willebrand factor propeptide in vascular disorders: A tool to distinguish between acute and chronic endothelial cell perturbation. Blood.

[22] Mancini I., Baronciani L., Artoni A., Colpani P., Biganzoli M., Cozzi G., Novembrino C., Boscolo Anzoletti M., De Zan V., Pagliari M.T. (2021). The ADAMTS13-von Willebrand factor axis in COVID-19 patients. J. Thromb. Haemost..

[23] Zhang L., Yan X., Fan Q., Liu H., Liu X., Liu Z., Zhang Z. (2020). D-dimer levels on admission to predict in-hospital mortality in patients with Covid-19. J. Thromb. Haemost..

[24] Demelo-Rodríguez P., Cervilla-Muñoz E., Ordieres-Ortega L., Parra-Virto A., Toledano-Macías M., Toledo-Samaniego N., García-García A., García-Fernández-Bravo I., Ji Z., De-Miguel-Diez J. (2020). Incidence of asymptomatic deep vein thrombosis in patients with COVID-19 pneumonia and elevated D-dimer levels. Thromb. Res..

[25] Simadibrata D.M., Lubis A.M. (2020). D-dimer levels on admission and all-cause mortality risk in COVID-19 patients: A meta-analysis. Epidemiol. Infect..

[26] Sakka M., Connors J.M., Hékimian G., Martin-Toutain I., Crichi B., Colmegna I., Bonnefont-Rousselot D., Farge D., Frere C. (2020). Association between D-Dimer levels and mortality in patients with coronavirus disease 2019 (COVID-19): A systematic review and pooled analysis. JMV J. Méd. Vasc..

[27] Yanagisawa M., Kurihara H., Kimura S., Tomobe Y., Kobayashi M., Mitsui Y., Yazaki Y., Goto K., Masaki T. (1988). A novel potent vasoconstrictor peptide produced by vascular endothelial cells. Nature.

[28] Mitaka C., Hirata Y., Nagura T., Tsunoda Y., Amaha K. (1993). Circulating endothelin-1 concentrations in acute respiratory failure. Chest.

[29] Albertini M., Clement M.G., Hussain S.N.A. (2003). Role of endothelin ETA receptors in sepsis-induced mortality, vascular leakage, and tissue injury in rats. Eur. J. Pharmacol..

[30] Rossi P., Wanecek M., Konrad D., Oldner A. (2004). Tezosentan Counteracts Endotoxin-Induced Pulmonary Edema and Improves Gas Exchange. Shock.

[31] Nakano Y., Tasaka S., Saito F., Yamada W., Shiraishi Y., Ogawa Y., Koh H., Hasegawa N., Fujishima S., Hashimoto S. (2007). Endothelin-1 level in epithelial lining fluid of patients with acute respiratory distress syndrome. Respirology.

[32] Forni M., Mazzola S., Ribeiro L.A., Pirrone F., Zannoni A., Bernardini C., Bacci M.L., Albertini M. (2005). Expression of endothelin-1 system in a pig model of endotoxic shock. Regul. Pept..

[33] Carpenter T.C., Stenmark K.R. (2000). Endothelin receptor blockade decreases lung water in young rats exposed to viral infection and hypoxia. Am. J. Physiol. Lung Cell Mol. Physiol..

[34] Fujii Y., Magder S., Cernacek P., Goldberg P., Guo Y. (2000). Endothelin Receptor Blockade Attenuates Lipopolysaccharide-induced Pulmonary Nitric Oxide Production. Am. J. Respir. Crit. Care Med..

[35] Patel S., Liu X., Liu M., Stephani R., Patel H., Cantor J. (2014). HJP272, A novel endothelin receptor antagonist, attenuates lipopolysaccharide-induced acute lung injury in hamsters. Lung.

[36] Trabold B., Pawlik M., Nietsch R., Bitzinger D.I., Gruber M., Ittner K.P., Lubnow M. (2009). Bosentan reduces oxidative burst in acid aspiration-induced lung injury in rats. Injury.

[37] Zhang Z., Jian X., Zhang W., Wang J., Zhou Q. (2013). Using Bosentan to Treat Paraquat Poisoning-Induced Acute Lung Injury in Rats. PLoS ONE.

[38] Guo Q., Huang J.A., Fraidenburg D.R. (2014). Bosentan as Rescue Treatment in Refractory Hypoxemia and Pulmonary Hypertension in a Patient with ARDS and H7N9 Influenza Virus Infection. Lung.

[39] Choi W.I., Quinn D.A., Park K.M., Moufarrej R.K., Jafari B., Syrkina O., Bonventre J.V., Hales C.A. (2003). Systemic microvascular leak in an in vivo rat model of ventilator-induced lung injury. Am. J. Respir. Crit. Care Med..

[40] Kubes P. (1995). Nitric Oxide Affects Microvascular Permeability in the Intact and Inflamed Vasculature. Microcirculation.

[41] Kristof A.S., Goldberg P., Laubach V., Hussain S.N.A. (1998). Role of inducible nitric oxide synthase in endotoxin-induced acute lung injury. Am. J. Respir. Crit. Care Med..

[42] Takenaka K., Nishimura Y., Nishiuma T., Sakashita A., Yamashita T., Kobayashi K., Satouchi M., Ishida T., Kawashima S., Yokoyama M. (2006). Ventilator-induced lung injury is reduced in transgenic mice that overexpress endothelial nitric oxide synthase. Am. J. Physiol. Lung Cell. Mol. Physiol..

[43] Förstermann U., Münzel T. (2006). Endothelial nitric oxide synthase in vascular disease: From marvel to menace. Circulation.

[44] Bernstein K.E., Khan Z., Giani J.F., Cao D.Y., Bernstein E.A., Shen X.Z. (2018). Angiotensin-converting enzyme in innate and adaptive immunity. Nat. Rev. Nephrol..

[45] Benigni A., Cassis P., Remuzzi G. (2010). Angiotensin II revisited: New roles in inflammation, immunology and aging. EMBO Mol. Med..

[46] Mateo T., Naim Abu Nabah Y., Abu Taha M., Mata M., Cerdá-Nicolás M., Proudfoot A.E.I., Stahl R.A.K., Issekutz A.C., Cortijo J., Morcillo E.J. (2006). Angiotensin II-Induced Mononuclear Leukocyte Interactions with Arteriolar and Venular Endothelium Are Mediated by the Release of Different CC Chemokines. J. Immunol..

[47] Ciceri F., Beretta L., Scandroglio A.M., Colombo S., Landoni G., Ruggeri A., Peccatori J., D’Angelo A., De Cobelli F., Rovere-Querini P. (2020). Microvascular COVID-19 lung vessels obstructive thromboinflammatory syndrome (MicroCLOTS): An atypical acute respiratory distress syndrome working hypothesis. Crit. Care Resusc..

[48] Grillet F., Behr J., Calame P., Aubry S., Delabrousse E. (2020). Acute Pulmonary Embolism Associated with COVID-19 Pneumonia Detected with Pulmonary CT Angiography. Radiology.

[49] Bikdeli B., Madhavan M.V., Jimenez D., Chuich T., Dreyfus I., Driggin E., Der Nigoghossian C., Ageno W., Madjid M., Guo Y. (2020). COVID-19 and Thrombotic or Thromboembolic Disease: Implications for Prevention, Antithrombotic Therapy, and Follow-Up. J. Am. Coll. Cardiol..

[50] Chen C., Zhou Y., Wang D.W. (2020). SARS-CoV-2: A potential novel etiology of fulminant myocarditis. Herz.

[51] Huang C., Wang Y., Li X., Ren L., Zhao J., Hu Y., Zhang L., Fan G., Xu J., Gu X. (2020). Clinical features of patients infected with 2019 novel coronavirus in Wuhan, China. Lancet.

[52] Wang D., Hu B., Hu C., Zhu F., Liu X., Zhang J., Wang B., Xiang H., Cheng Z., Xiong Y. (2020). Clinical Characteristics of 138 Hospitalized Patients with 2019 Novel Coronavirus-Infected Pneumonia in Wuhan, China. JAMA J. Am. Med. Assoc..

[53] Yang X., Yu Y., Xu J., Shu H., Xia J., Liu H., Wu Y., Zhang L., Yu Z., Fang M. (2020). Clinical course and outcomes of critically ill patients with SARS-CoV-2 pneumonia in Wuhan, China: A single-centered, retrospective, observational study. Lancet Respir. Med..

[54] Rodriguez-Morales A.J., Cardona-Ospina J.A., Gutiérrez-Ocampo E., Villamizar-Peña R., Holguin-Rivera Y., Escalera-Antezana J.P., Alvarado-Arnez L.E., Bonilla-Aldana D.K., Franco-Paredes C., Henao-Martinez A.F. (2020). Clinical, laboratory and imaging features of COVID-19: A systematic review and meta-analysis. Travel Med. Infect. Dis..

[55] Lippi G., Plebani M. (2020). Laboratory abnormalities in patients with COVID-2019 infection. Clin. Chem. Lab. Med..

[56] Li X., Wang L., Yan S., Yang F., Xiang L., Zhu J., Shen B., Gong Z. (2020). Clinical characteristics of 25 death cases with COVID-19: A retrospective review of medical records in a single medical center, Wuhan, China. Int. J. Infect. Dis..

[57] Zhang B., Zhou X., Qiu Y., Song Y., Feng F., Feng J., Song Q., Jia Q., Wang J. (2020). Clinical characteristics of 82 cases of death from COVID-19. PLoS ONE.

[58] Shi S., Qin M., Shen B., Cai Y., Liu T., Yang F., Gong W., Liu X., Liang J., Zhao Q. (2020). Association of Cardiac Injury with Mortality in Hospitalized Patients with COVID-19 in Wuhan, China. JAMA Cardiol..

[59] Madjid M., Miller C.C., Zarubaev V.V., Marinich I.G., Kiselev O.I., Lobzin Y.V., Filippov A.E., Casscells S.W. (2007). Influenza epidemics and acute respiratory disease activity are associated with a surge in autopsy-confirmed coronary heart disease death: Results from 8 years of autopsies in 34 892 subjects. Eur. Heart J..

[60] Nguyen J.L., Yang W., Ito K., Matte T.D., Shaman J., Kinney P.L. (2016). Seasonal influenza infections and cardiovascular disease mortality. JAMA Cardiol..

[61] Wu C., Hu X., Song J., Du C., Xu J., Yang D., Chen D., Zhong M., Jiang J., Xiong W. Heart injury signs are associated with higher and earlier mortality in coronavirus disease 2019 (COVID-19). medRxiv.

[62] Chen G., Wu D., Guo W., Cao Y., Huang D., Wang H., Wang T., Zhang X., Chen H., Yu H. (2020). Clinical and immunological features of severe and moderate coronavirus disease 2019. J. Clin. Investig..

[63] Riphagen S., Gomez X., Gonzalez-Martinez C., Wilkinson N., Theocharis P. (2020). Hyperinflammatory shock in children during COVID-19 pandemic. Lancet.

[64] Jones V.G., Mills M., Suarez D., Hogan C.A., Yeh D., Bradley Segal J., Nguyen E.L., Barsh G.R., Maskatia S., Mathew R. (2020). COVID-19 and Kawasaki Disease: Novel Virus and Novel Case. Hosp. Pediatr..

[65] Whittaker E., Bamford A., Kenny J., Kaforou M., Jones C.E., Shah P., Ramnarayan P., Fraisse A., Miller O., Davies P. (2020). Clinical Characteristics of 58 Children with a Pediatric Inflammatory Multisystem Syndrome Temporally Associated with SARS-CoV-2. JAMA J. Am. Med. Assoc..

[66] Baig A.M., Khaleeq A., Ali U., Syeda H. (2020). Evidence of the COVID-19 Virus Targeting the CNS: Tissue Distribution, Host-Virus Interaction, and Proposed Neurotropic Mechanisms. ACS Chem. Neurosci..

[67] Palasca O., Santos A., Stolte C., Gorodkin J., Jensen L.J. (2018). TISSUES 2.0: An integrative web resource on mammalian tissue expression. Database.

[68] Paniz-Mondolfi A., Bryce C., Grimes Z., Gordon R.E., Reidy J., Lednicky J., Sordillo E.M., Fowkes M. (2020). Central nervous system involvement by severe acute respiratory syndrome coronavirus-2 (SARS-CoV-2). J. Med. Virol..

[69] Aragão M.F.V.V., Leal M.C., Cartaxo Filho O.Q., Fonseca T.M., Valença M.M. (2020). Anosmia in COVID-19 associated with injury to the olfactory bulbs evident on MRI. Am. J. Neuroradiol..

[70] Whitcroft K.L., Hummel T. (2020). Olfactory Dysfunction in COVID-19: Diagnosis and Management. JAMA J. Am. Med. Assoc..

[71] Butowt R., Bilinska K. (2020). SARS-CoV-2: Olfaction, Brain Infection, and the Urgent Need for Clinical Samples Allowing Earlier Virus Detection. ACS Chem. Neurosci..

[72] Coolen T., Lolli V., Sadeghi N., Rovai A., Trotta N., Taccone F.S., Creteur J., Henrard S., Goffard J.C., Dewitte O. (2020). Early postmortem brain MRI findings in COVID-19 non-survivors. Neurology.

[73] Jakhmola S., Indari O., Chatterjee S., Jha H.C. (2020). SARS-CoV-2, an Underestimated Pathogen of the Nervous System. SN Compr. Clin. Med..

[74] Mao L., Jin H., Wang M., Hu Y., Chen S., He Q., Chang J., Hong C., Zhou Y., Wang D. (2020). Neurologic Manifestations of Hospitalized Patients with Coronavirus Disease 2019 in Wuhan, China. JAMA Neurol..

[75] Li Y., Li M., Wang M., Zhou Y., Chang J., Xian Y., Wang D., Mao L., Jin H., Hu B. (2020). Acute cerebrovascular disease following COVID-19: A single center, retrospective, observational study. Stroke Vasc. Neurol..

[76] Beyrouti R., Adams M.E., Benjamin L., Cohen H., Farmer S.F., Goh Y.Y., Humphries F., Jäger H.R., Losseff N.A., Perry R.J. (2020). Characteristics of ischaemic stroke associated with COVID-19. J. Neurol. Neurosurg. Psychiatry.

[77] Avula A., Nalleballe K., Narula N., Sapozhnikov S., Dandu V., Toom S., Glaser A., Elsayegh D. (2020). COVID-19 presenting as stroke. Brain. Behav. Immun..

[78] Guo T., Fan Y., Chen M., Wu X., Zhang L., He T., Wang H., Wan J., Wang X., Lu Z. (2020). Cardiovascular Implications of Fatal Outcomes of Patients with Coronavirus Disease 2019 (COVID-19). JAMA Cardiol..

[79] Pennisi M., Lanza G., Falzone L., Fisicaro F., Ferri R., Bella R. (2020). Sars-cov-2 and the nervous system: From clinical features to molecular mechanisms. Int. J. Mol. Sci..

[80] Yachou Y., El Idrissi A., Belapasov V., Ait Benali S. (2020). Neuroinvasion, neurotropic, and neuroinflammatory events of SARS-CoV-2: Understanding the neurological manifestations in COVID-19 patients. Neurol. Sci..

[81] Helbok R., Chou S.H.Y., Beghi E., Mainali S., Frontera J., Robertson C., Fink E., Schober M., Moro E., McNett M. (2020). NeuroCOVID: It’s time to join forces globally. Lancet Neurol..

[82] Guan W., Ni Z., Hu Y., Liang W., Ou C., He J., Liu L., Shan H., Lei C., Hui D.S.C. (2020). Clinical Characteristics of Coronavirus Disease 2019 in China. N. Engl. J. Med..

[83] Cheng Y., Luo R., Wang K., Zhang M., Wang Z., Dong L., Li J., Yao Y., Ge S., Xu G. (2020). Kidney disease is associated with in-hospital death of patients with COVID-19. Kidney Int..

[84] Yang X., Tian S., Guo H. (2020). Acute kidney injury and renal replacement therapy in COVID-19 patients: A systematic review and meta-analysis. Int. Immunopharmacol..

[85] Joob B., Wiwanitkit V. (2014). Novel Middle East respiratory syndrome and renal failure. Ren. Fail..

[86] Hamming I., Timens W., Bulthuis M.L.C., Lely A.T., Navis G.J., van Goor H. (2004). Tissue distribution of ACE2 protein, the functional receptor for SARS coronavirus. A first step in understanding SARS pathogenesis. J. Pathol..

[87] Lely A.T., Hamming I., van Goor H., Navis G.J. (2004). Renal ACE2 expression in human kidney disease. J. Pathol..

[88] Kamilic J., Hamming I., Kreutz R., Bolbrinker J., Siems W.E., Nassar I., Sluimer J.C., Walther T., Navis G.J., Van Goor H. (2010). Renal ACE2 expression and activity is unaltered during established hypertension in adult SHRSP and TGR(mREN2)27. Hypertens. Res..

[89] Giani J.F., Burghi V., Veiras L.C., Tomat A., Muñoz M.C., Cao G., Turyn D., Toblli J.E., Dominici F.P. (2012). Angiotensin-(1-7) attenuates diabetic nephropathy in Zucker diabetic fatty rats. Am. J. Physiol. Ren. Physiol..

[90] Bae E.H., Konvalinka A., Fang F., Zhou X., Williams V., Maksimowski N., Song X., Zhang S.L., John R., Oudit G.Y. (2015). Characterization of the intrarenal renin-angiotensin system in experimental Alport syndrome. Am. J. Pathol..

[91] Cao G., Della Penna S.L., Kouyoumdzian N.M., Choi M.R., Gorzalczany S., Fernández B.E., Toblli J.E., Rosón M.I. (2017). Immunohistochemical expression of intrarenal renin angiotensin system components in response to tempol in rats fed a high salt diet. World J. Nephrol..

[92] Errarte P., Beitia M., Perez I., Manterola L., Lawrie C.H., Solano-Iturri J.D., Calvete-Candenas J., Unda M., López J.I., Larrinaga G. (2017). Expression and activity of angiotensin-regulating enzymes is associated with prognostic outcome in clear cell renal cell carcinoma patients. PLoS ONE.

[93] Yalameha B., Roshan B., Bhaskar L.V.K.S., Mohmoodnia L. (2020). Perspectives on the relationship of renal disease and coronavirus disease 2019. J. Nephropharmacol..

[94] Diao B., Wang C., Wang R., Feng Z., Tan Y., Wang H., Wang C., Liu L., Liu Y., Liu Y. (2020). Human Kidney is a Target for Novel Severe Acute Respiratory Syndrome Coronavirus 2 (SARS-CoV-2) Infection. medRxiv.

[95] Zhou F., Yu T., Du R., Fan G., Liu Y., Liu Z., Xiang J., Wang Y., Song B., Gu X. (2020). Clinical course and risk factors for mortality of adult inpatients with COVID-19 in Wuhan, China: A retrospective cohort study. Lancet.

[96] Richardson S., Hirsch J.S., Narasimhan M., Crawford J.M., McGinn T., Davidson K.W., Barnaby D.P., Becker L.B., Chelico J.D., Cohen S.L. (2020). Presenting Characteristics, Comorbidities, and Outcomes Among 5700 Patients Hospitalized With COVID-19 in the New York City Area. JAMA.

[97] Li J., Fan J.G. (2020). Characteristics and mechanism of liver injury in 2019 coronavirus disease. J. Clin. Transl. Hepatol..

[98] Kerr R., Newsome P., Germain L., Thomson E., Dawson P., Stirling D., Ludlam C.A. (2003). Effects of acute liver injury on blood coagulation. J. Thromb. Haemost..

[99] Gencer S., Lacy M., Atzler D., Van Der Vorst E.P.C., Döring Y., Weber C. (2020). Immunoinflammatory, Thrombohaemostatic, and Cardiovascular Mechanisms in COVID-19. Thromb. Haemost..

[100] Bavishi C., Maddox T.M., Messerli F.H. (2020). Coronavirus Disease 2019 (COVID-19) Infection and Renin Angiotensin System Blockers. JAMA Cardiol..

[101] Buckley L.F., Cheng J.W.M., Desai A. (2020). Cardiovascular Pharmacology in the Time of COVID-19: A Focus on Angiotensin-Converting Enzyme 2. J. Cardiovasc. Pharmacol..

[102] Danser A.H.J., Epstein M., Batlle D. (2020). Renin-Angiotensin System Blockers and the COVID-19 Pandemic: At Present There Is No Evidence to Abandon Renin-Angiotensin System Blockers. Hypertension.

[103] Kreutz R., Algharably E.A.E.H., Azizi M., Dobrowolski P., Guzik T., Januszewicz A., Persu A., Prejbisz A., Riemer T.G., Wang J.G. (2020). Hypertension, the renin-angiotensin system, and the risk of lower respiratory tract infections and lung injury: Implications for covid-19. Cardiovasc. Res..

[104] Matsuzawa Y., Ogawa H., Kimura K., Konishi M., Kirigaya J., Fukui K., Tsukahara K., Shimizu H., Iwabuchi K., Yamada Y. (2020). Renin–angiotensin system inhibitors and the severity of coronavirus disease 2019 in Kanagawa, Japan: A retrospective cohort study. Hypertens. Res..

[105] Rico-Mesa J.S., White A., Anderson A.S. (2020). Outcomes in Patients with COVID-19 Infection Taking ACEI/ARB. Curr. Cardiol. Rep..

[106] Zhang P., Zhu L., Cai J., Lei F., Qin J.J., Xie J., Liu Y.M., Zhao Y.C., Huang X., Lin L. (2020). Association of Inpatient Use of Angiotensin-Converting Enzyme Inhibitors and Angiotensin II Receptor Blockers with Mortality among Patients with Hypertension Hospitalized with COVID-19. Circ. Res..

[107] Tipnis S.R., Hooper N.M., Hyde R., Karran E., Christie G., Turner A.J. (2000). A human homolog of angiotensin-converting enzyme: Cloning and functional expression as a captopril-insensitive carboxypeptidase. J. Biol. Chem..

[108] Donoghue M., Hsieh F., Baronas E., Godbout K., Gosselin M., Stagliano N., Donovan M., Woolf B., Robison K., Jeyaseelan R. (2000). A novel angiotensin-converting enzyme—Related to angiotensin 1-9. Circ. Res..

[109] Fang L., Karakiulakis G., Roth M. (2020). Are patients with hypertension and diabetes mellitus at increased risk for COVID-19 infection?. Lancet Respir. Med..

[110] Brady N.R., Hamacher-Brady A., Westerhoff H.V., Gottlieb R.A. (2006). A wave of reactive oxygen species (ROS)-induced ROS release in a sea of excitable mitochondria. Antioxid. Redox Signal..

[111] Zorov D.B., Juhaszova M., Sollott S.J. (2006). Mitochondrial ROS-induced ROS release: An update and review. Biochim. Biophys. Acta Bioenerg..

[112] Zhang G.X., Lu X.M., Kimura S., Nishiyama A. (2007). Role of mitochondria in angiotensin II-induced reactive oxygen species and mitogen-activated protein kinase activation. Cardiovasc. Res..

[113] Shahin Y., Khan J.A., Samuel N., Chetter I. (2011). Angiotensin converting enzyme inhibitors effect on endothelial dysfunction: A meta-analysis of randomised controlled trials. Atherosclerosis.

[114] Li S., Wu Y., Yu G., Xia Q., Xu Y. (2014). Angiotensin II receptor blockers improve peripheral endothelial function: A meta-analysis of randomized controlled trials. PLoS ONE.

[115] Imai Y., Kuba K., Rao S., Huan Y., Guo F., Guan B., Yang P., Sarao R., Wada T., Leong-Poi H. (2005). Angiotensin-converting enzyme 2 protects from severe acute lung failure. Nature.

[116] Gautret P., Lagier J.C., Parola P., Hoang V.T., Meddeb L., Mailhe M., Doudier B., Courjon J., Giordanengo V., Vieira V.E. (2020). Hydroxychloroquine and azithromycin as a treatment of COVID-19: Results of an open-label non-randomized clinical trial. Int. J. Antimicrob. Agents.

[117] Lagier J., Million M., Gautret P., Colson P., Cortaredona S., Giraud-Gatineau A., Honoré S., Gaubert J., Fournier P., Tissot-Dupont H. (2020). Outcomes of 3,737 COVID-19 patients treated with hydroxychloroquine/azithromycin and other regimens in Marseille, France: A retrospective analysis. Travel Med. Infect. Dis..

[118] Chen X., Zhang Y., Zhu B., Zeng J., Hong W., He X., Chen J., Zheng H., Qiu S., Deng Y. (2020). Associations of clinical characteristics and antiviral drugs with viral RNA clearance in patients with COVID-19 in Guangzhou, China: A retrospective cohort study. medRxiv.

[119] Mallat J., Hamed F., Balkis M., Mohamed M.A., Mooty M., Malik A., Nusair A., Bonilla M.F. (2020). Hydroxychloroquine is associated with slower viral clearance in clinical COVID-19 patients with mild to moderate disease. Medicine.

[120] Huang M., Li M., Xiao F., Pang P., Liang J., Tang T., Liu S., Chen B., Shu J., You Y. (2020). Preliminary evidence from a multicenter prospective observational study of the safety and efficacy of chloroquine for the treatment of COVID-19. Natl. Sci. Rev..

[121] Huang M., Tang T., Pang P., Li M., Ma R., Lu J., Shu J., You Y., Chen B., Liang J. (2020). Treating COVID-19 with Chloroquine. J. Mol. Cell Biol..

[122] Tang W., Cao Z., Han M., Wang Z., Chen J., Sun W., Wu Y., Xiao W., Liu S., Chen E. (2020). Hydroxychloroquine in patients with mainly mild to moderate coronavirus disease 2019: Open label, randomised controlled trial. BMJ.

[123] FDA (2020). Letter Revoking EUA for Chloroquine Phosphate and Hydroxychloroquine Sulfate.

[124] Michaud V., Dow P., Al Rihani S.B., Deodhar M., Arwood M., Cicali B., Turgeon J. (2020). Risk Assessment of Drug-Induced Long QT Syndrome for Some COVID-19 Repurposed Drugs. Clin. Transl. Sci..

[125] Saleh M., Gabriels J., Chang D., Soo Kim B., Mansoor A., Mahmood E., Makker P., Ismail H., Goldner B., Willner J. (2020). Effect of Chloroquine, Hydroxychloroquine, and Azithromycin on the Corrected QT Interval in Patients with SARS-CoV-2 Infection. Circ. Arrhythmia Electrophysiol..

[126] Deftereos S.G., Giannopoulos G., Vrachatis D.A., Siasos G.D., Giotaki S.G., Gargalianos P., Metallidis S., Sianos G., Baltagiannis S., Panagopoulos P. (2020). Effect of Colchicine vs. Standard Care on Cardiac and Inflammatory Biomarkers and Clinical Outcomes in Patients Hospitalized With Coronavirus Disease 2019: The GRECCO-19 Randomized Clinical Trial. JAMA Netw. Open.

[127] The Montreal Heart Institute Press Release: Colchicine Reduces the Risk of COVID-19-Related Complications. https://app.cyberimpact.com/newsletter-view-online?ct=guhsMu_jogsWK5zuKuZWMiFdWXxrNhn6Nkcjb1fm-HUAuS81ZbwD0N6bKX9bJ23ALFDAfrG83CWBnSzT41zxRA.

[128] Arabi Y.M., Mandourah Y., Al-Hameed F., Sindi A.A., Almekhlafi G.A., Hussein M.A., Jose J., Pinto R., Al-Omari A., Kharaba A. (2018). Corticosteroid therapy for critically ill patients with middle east respiratory syndrome. Am. J. Respir. Crit. Care Med..

[129] Stockman L.J., Bellamy R., Garner P. (2006). SARS: Systematic review of treatment effects. PLoS Med..

[130] (2020). The RECOVERY Collaborative Group Dexamethasone in Hospitalized Patients with Covid-19—Preliminary Report. N. Engl. J. Med..

[131] (2020). The RECOVERY Collaborative Group Effect of Hydroxychloroquine in Hospitalized Patients with Covid-19. N. Engl. J. Med..

[132] Garvin M.R., Alvarez C., Miller J.I., Prates E.T., Walker A.M., Amos B.K., Mast A.E., Justice A., Aronow B., Jacobson D. (2020). A mechanistic model and therapeutic interventions for COVID-19 involving a RAS-mediated bradykinin storm. Elife.

[133] Kong J., Zhu X., Shi Y., Liu T., Chen Y., Bhan I., Zhao Q., Thadhani R., Chun Li Y. (2013). VDR attenuates acute lung injury by blocking Ang-2-Tie-2 pathway and renin-angiotensin system. Mol. Endocrinol..

[134] Xu J., Yang J., Chen J., Luo Q., Zhang Q., Zhang H. (2017). Vitamin D alleviates lipopolysaccharide-induced acute lung injury via regulation of the renin-angiotensin system. Mol. Med. Rep..

[135] Murai I.H., Fernandes A.L., Sales L.P., Pinto A.J., Goessler K.F., Duran C.S.C., Silva C.B.R., Franco A.S., Macedo M.B., Dalmolin H.H.H. (2021). Effect of a Single High Dose of Vitamin D 3 on Hospital Length of Stay in Patients With Moderate to Severe COVID-19. JAMA.

[136] Leaf D.E., Ginde A.A. (2021). Vitamin D 3 to Treat COVID-19. JAMA.

[137] De Maat S., De Mast Q., Danser A.H.J., Van De Veerdonk F.L., Maas C. (2020). Impaired Breakdown of Bradykinin and Its Metabolites as a Possible Cause for Pulmonary Edema in COVID-19 Infection. Semin. Thromb. Hemost..

[138] Sodhi C.P., Wohlford-Lenane C., Yamaguchi Y., Prindle T., Fulton W.B., Wang S., McCray P.B., Chappell M., Hackam D.J., Jia H. (2018). Attenuation of pulmonary ACE2 activity impairs inactivation of des-arg9 bradykinin/BKB1R axis and facilitates LPS-induced neutrophil infiltration. Am. J. Physiol. Lung Cell. Mol. Physiol..

[139] Lindsey C.J., Nakaie C.R., Martins D.T.O. (1989). Central nervous system kinin receptors and the hypertensive response mediated by bradykinin. Br. J. Pharmacol..

[140] Hess R., Wujak L., Hesse C., Sewald K., Jonigk D., Warnecke G., Fieguth H.G., De Maat S., Maas C., Bonella F. (2017). Coagulation factor XII regulates inflammatory responses in human lungs. Thromb. Haemost..

[141] Qadri F., Bader M. (2018). Kinin B1 receptors as a therapeutic target for inflammation. Expert Opin. Ther. Targets.

[142] van de Veerdonk F.L., Kouijzer I.J.E., de Nooijer A.H., van der Hoeven H.G., Maas C., Netea M.G., Brüggemann R.J.M. (2020). Outcomes Associated With Use of a Kinin B2 Receptor Antagonist Among Patients With COVID-19. JAMA Netw. Open.

[143] EPAR Summary for the Public: Firazyr Icatibant. https://www.ema.europa.eu/en/documents/overview/firazyr-epar-summary-public_en.pdf.

[144] Mansour E., Bueno F.F., de Lima-Júnior J.C., Palma A., Monfort-Pires M., Bombassaro B., Araujo E.P., Bernardes A.F., Ulaf R.G., Nunes T.A. (2021). Evaluation of the efficacy and safety of icatibant and C1 esterase/kallikrein inhibitor in severe COVID-19: Study protocol for a three-armed randomized controlled trial. Trials.

[145] Phillip J.T., Robert A.B. (2012). Depression, anxiety, and cardiac morbidity outcomes after coronary artery bypass surgery: A contemporary and practical review. J. Geriatr. Cardiol..

[146] Michaud V., Deodhar M., Arwood M., Al Rihani S.B., Dow P., Turgeon J. (2020). ACE2 as a Therapeutic Target for COVID-19; Its Role in Infectious Processes and Regulation by Modulators of the RAAS System. J. Clin. Med..

[147] Rubin D.B. (2006). Basic Concepts of Statistical Inference for Causal Effects in Experiments and Observational Studies.

[148] Boutron I., Chaimani A., Devane D., Meerpohl J.J., Rada G., Hróbjartsson A., Tovey D., Grasselli G., Ravaud P. (2020). Interventions for the treatment of COVID-19: A living network meta-analysis. Cochrane Database Syst. Rev..

[149] Vandvik P.O., Brignardello-Petersen R., Guyatt G.H. (2016). Living cumulative network meta-analysis to reduce waste in research: A paradigmatic shift for systematic reviews?. BMC Med..

[150] Nikolakopoulou A., Mavridis D., Egger M., Salanti G. (2018). Continuously updated network meta-analysis and statistical monitoring for timely decision-making. Stat. Methods Med. Res..

[151] Créquit P., Trinquart L., Yavchitz A., Ravaud P. (2016). Wasted research when systematic reviews fail to provide a complete and up-to-date evidence synthesis: The example of lung cancer. BMC Med..

